# Poly[[(μ_2_-di-3-pyridyl­methanone-κ^2^ *N*:*N*′)(μ_2_-hexa­fluoro­silicato-κ^2^ *F*:*F*′)copper(II)] dihydrate]

**DOI:** 10.1107/S1600536812002267

**Published:** 2012-01-31

**Authors:** Yong-Li Yang

**Affiliations:** a Department of Chemistry, Capital Normal University, Beijing 100048, People’s Republic of China

## Abstract

In the title complex, {[Cu(SiF_6_)(C_11_H_8_N_2_O)_2_]·2H_2_O}_*n*_, the Cu^II^ atom adopts an N_4_F_2_-octa­hedral coordination geometry with four pyridine N atoms in the equatorial sites and two F atoms in the axial sites. The di-3-pyridyl­methanone and hexa­fluoro­silicate ligands act as bidentate ligands, linking symmetry-related Cu^II^ atoms. Water mol­ecules form O—H⋯O and O—H⋯F hydrogen bonds with the di-3-pyridyl­methanone and hexa­fluoro­silicate ligands. The Cu^2+^ and SiF_6_^2−^ ions are each located on a twofold axis.

## Related literature

For background to the coordination chemistry of pyridyl-based derivatives, see: Manriquez *et al.* (1991 ▶); Wang *et al.* (2009 ▶). For dipyridyl­methanone, see: Boudalis *et al.* (2003 ▶). For transition metal complexes of di-3-pyridyl­methanone, see: Chen *et al.* (2005 ▶, 2009 ▶); Chen & Mak (2005 ▶).
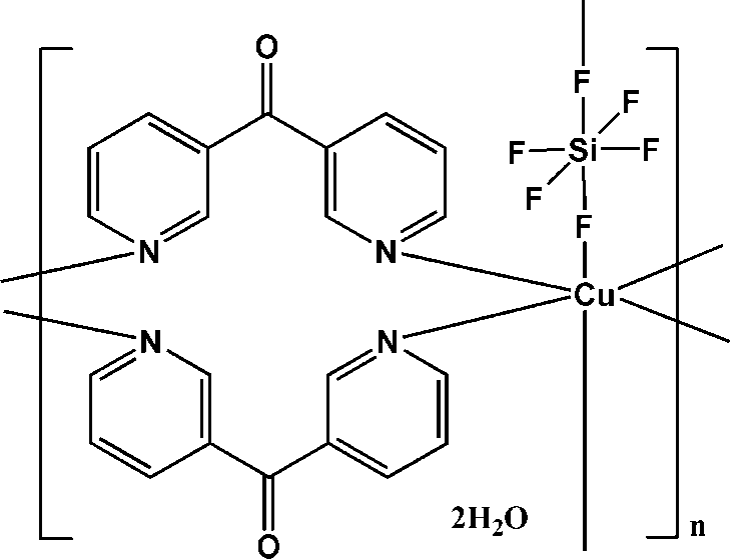


## Experimental

### 

#### Crystal data

[Cu(SiF_6_)(C_11_H_8_N_2_O)_2_]·2H_2_O
*M* *_r_* = 610.05
Monoclinic, 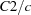

*a* = 22.276 (3) Å
*b* = 8.0625 (11) Å
*c* = 15.773 (2) Å
β = 123.757 (2)°
*V* = 2355.2 (5) Å^3^
*Z* = 4
Mo *K*α radiation
μ = 1.07 mm^−1^
*T* = 296 K
0.40 × 0.32 × 0.30 mm

#### Data collection

Bruker SMART APEXII CCD area-detector’ diffractometer
Absorption correction: multi-scan (*SADABS*; Bruker, 2007 ▶) *T*_min_ = 0.742, *T*_max_ = 1.000
6195 measured reflections
2082 independent reflections
1822 reflections with *I* > 2σ(*I*)
*R*_int_ = 0.031

#### Refinement

*R*[*F* ^2^ > 2σ(*F* ^2^)] = 0.034
*wR*(*F* ^2^) = 0.094
*S* = 1.02
2082 reflections
174 parameters
H-atom parameters constrained
Δρ_max_ = 0.54 e Å^−3^
Δρ_min_ = −0.31 e Å^−3^

### 

Data collection: *APEX2* (Bruker, 2007 ▶); cell refinement: *APEX2* and *SAINT* (Bruker, 2007 ▶); data reduction: *SAINT*; program(s) used to solve structure: *SHELXS97* (Sheldrick, 2008 ▶); program(s) used to refine structure: *SHELXL97* (Sheldrick, 2008 ▶); molecular graphics: *SHELXTL* (Sheldrick, 2008 ▶); software used to prepare material for publication: *SHELXTL* and *PLATON* (Spek, 2009 ▶).

## Supplementary Material

Crystal structure: contains datablock(s) I, global. DOI: 10.1107/S1600536812002267/aa2040sup1.cif

Structure factors: contains datablock(s) I. DOI: 10.1107/S1600536812002267/aa2040Isup2.hkl

Additional supplementary materials:  crystallographic information; 3D view; checkCIF report

## Figures and Tables

**Table 1:** Hydrogen-bond geometry (Å, °)

*D*—H⋯*A*	*D*—H	H⋯*A*	*D*⋯*A*	*D*—H⋯*A*
O1*W*—H1*B*⋯F1	0.89	1.89	2.777 (6)	173
O1*W*—H1*A*⋯O1^i^	0.89	2.03	2.850 (3)	153

Symmetry code: (i) 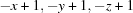.

**Fig. 1.**
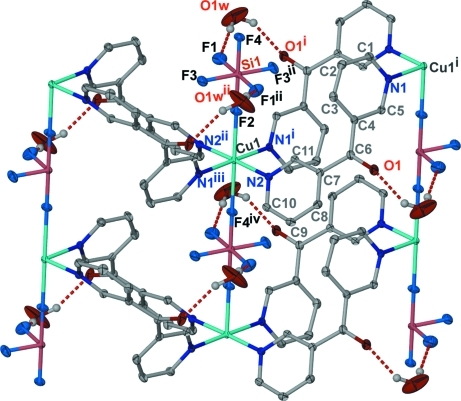
The title complex showing the atom-numbering scheme, with displacement ellipsoids shown at the 30% probability level. Hydrogen atoms are omitted for clarity. Symmetry codes: (i) -x+1, -y+1, -z+1; (ii) -x+1, y, -z+0.5; (iii) x, -y+1, z-0.5; (iv) x, y+1, z.

**Fig. 2.**
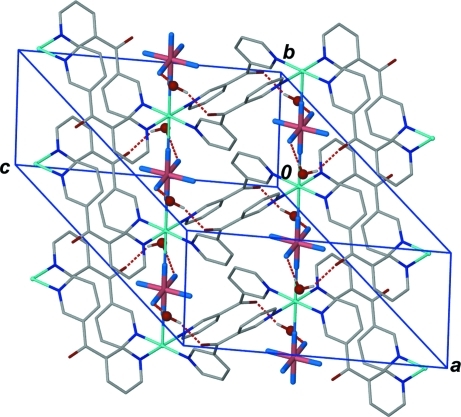
Lateral view of the two-dimensional framework in the bc plane of the title complex.The O atoms of lattice water are shown as red ball mode. All H atoms except water H atoms are omitted for clarity. The Si—F are shown in a thick bond mode. The red dashed lines represent H-bonding interactions.

**Fig. 3.**
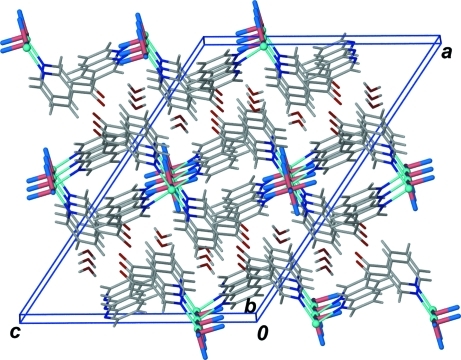
The packing diagram of the title complex.

## supplementary crystallographic information

### Comment

Pyridyl-based building blocks are widely used in construction various supramolecules of transition metal complexes (Manriquez *et al.* 1991; Wang *et al.*, 2009). Among them, dipyridylmethanone derivates are famous for their versatile linkage behavior in numbers of coordination supramolecular assemblies (Boudalis *et al.*, 2003). Di-3-pyridinylmethanone was provided to act as a flexible µ^2^-bridging mode in many coordination frameworks, such as one-dimensional helical and zigzag chains (Chen & Mak, 2005), two-dimensional nets (Chen *et al.*, 2005) as well as honeycomb-like three-dimensional frameworks (Chen *et al.*, 2009). Herein, we report a new structure derived from di-3-pyridinylmethanone, namely {[Cu(C_11_H_8_N_2_O)_2_SiF_6_]^.^2H_2_O}_n_.

In the title complex, the Cu^II^ atom adopts an N4F2-octahedral coordination geometry with four pyridyl N atoms at the equatorial sites and two F atoms at the axial sites (Fig. 1). The di-3-pyridylmethanone and hexafluorosilicate ligands act as bidentate ligands linking symmetry-related Cu^II^ atoms. Water molecules form hydrogen bonds with di-3-pyridylmethanone and hexafluorosilicate ligands bridging them together (Table 1). Cu^2+^ and SiF_6_^2-^ ions are located on a twofold axis, see Fig. 2 and Fig. 3. The structure of the title complex is remarkable different from a similar complex [(Cu*L*_2_)(BF_4_)_2_]_n_ (*L* = di-3-pyridylmethanone, Chen *et al.* 2005), where the Cu^II^ adopts a square-plane N4-geometry with four ligands around the metal center, forming a (4,4) net structure.

### Experimental

The ligand was obtained according to the reported procedure (Chen & Mak, 2005). The Cu(BF_4_^-^)_2_^.^6H_2_O (35 mg, 0.1 mmol) and di-3-pyridylmethanone (38 mg, 0.2 mmol) were dissolved in a mixed solvent of 1ml methanol and 3 ml acetonitrile with stirring at room temperature. The (NH_4_)_2_SiF_6_ (18 mg, 0.1 mmol) was subsequently added to the solution. After 4 hours, the resulted clear solution was filtered and the filtrate was left to stay in air. The block crystals suitable for x-ray diffraction analysis were obtained after about one weak (29.9 mg, 49% yield).

### Refinement

All the H atoms were located in the difference electron density maps but were placed in idealized positions and allowed to ride on the carrier atoms, with C—H = 0.93 Å and with *U*_iso_(H) = 1.2*U*_eq_(C).

### Crystal data

**Table:**

[Cu(SiF_6_)(C_11_H_8_N_2_O)_2_]·2H_2_O	*F*(000) = 1236
*M**_r_* = 610.05	*D*_x_ = 1.720 Mg m^−^^3^
Monoclinic, *C*2/*c*	Mo *K*α radiation, λ = 0.71073 Å
Hall symbol: -C 2yc	Cell parameters from 245 reflections
*a* = 22.276 (3) Å	θ = 2.2–26.1°
*b* = 8.0625 (11) Å	µ = 1.07 mm^−^^1^
*c* = 15.773 (2) Å	*T* = 296 K
β = 123.757 (2)°	Block, blue
*V* = 2355.2 (5) Å^3^	0.40 × 0.32 × 0.30 mm
*Z* = 4	

### Data collection

**Table:**

Bruker SMART APEXII CCD area-detector' diffractometer	2082 independent reflections
Radiation source: fine-focus sealed tube	1822 reflections with *I* > 2σ(*I*)
graphite	*R*_int_ = 0.031
ω scans	θ_max_ = 25.0°, θ_min_ = 2.2°
Absorption correction: multi-scan (*SADABS*; Bruker, 2007)	*h* = −22→26
*T*_min_ = 0.742, *T*_max_ = 1.000	*k* = −8→9
6195 measured reflections	*l* = −18→16

### Refinement

**Table:**

Refinement on *F*^2^	Primary atom site location: structure-invariant direct methods
Least-squares matrix: full	Secondary atom site location: difference Fourier map
*R*[*F*^2^ > 2σ(*F*^2^)] = 0.034	Hydrogen site location: inferred from neighbouring sites
*wR*(*F*^2^) = 0.094	H-atom parameters constrained
*S* = 1.02	*w* = 1/[σ^2^(*F*_o_^2^) + (0.0499*P*)^2^ + 3.641*P*] *P* = (*F*_o_^2^ + 2*F*_c_^2^)/3
2082 reflections	(Δ/σ)_max_ < 0.001
174 parameters	Δρ_max_ = 0.54 e Å^−^^3^
0 restraints	Δρ_min_ = −0.31 e Å^−^^3^

### Special details

**Table:**

Geometry. All s.u.'s (except the s.u. in the dihedral angle between two l.s. planes) are estimated using the full covariance matrix. The cell s.u.'s are taken into account individually in the estimation of s.u.'s in distances, angles and torsion angles; correlations between s.u.'s in cell parameters are only used when they are defined by crystal symmetry. An approximate (isotropic) treatment of cell s.u.'s is used for estimating s.u.'s involving l.s. planes.
Refinement. Refinement of F^2^ against ALL reflections. The weighted R-factor wR and goodness of fit S are based on F^2^, conventional R-factors R are based on F, with F set to zero for negative F^2^. The threshold expression of F^2^ > 2sigma(F^2^) is used only for calculating R-factors(gt) etc. and is not relevant to the choice of reflections for refinement. R-factors based on F^2^ are statistically about twice as large as those based on F, and R- factors based on ALL data will be even larger.

### Fractional atomic coordinates and isotropic or equivalent isotropic displacement parameters (Å^2^)

**Table:**

	*x*	*y*	*z*	*U*_iso_*/*U*_eq_	
Cu1	0.5000	0.74249 (4)	0.2500	0.02213 (16)	
F1	0.41305 (11)	0.2547 (2)	0.15265 (16)	0.0656 (6)	
F2	0.5000	0.4646 (2)	0.2500	0.0430 (6)	
F3	0.52519 (13)	0.25362 (17)	0.16853 (16)	0.0567 (6)	
F4	0.5000	0.0403 (2)	0.2500	0.0423 (5)	
O1	0.71275 (11)	0.6013 (2)	0.74098 (13)	0.0495 (5)	
N1	0.55932 (11)	0.2545 (2)	0.68811 (15)	0.0247 (4)	
N2	0.59150 (11)	0.7466 (2)	0.39361 (16)	0.0255 (4)	
C1	0.55661 (14)	0.1248 (3)	0.63175 (18)	0.0317 (5)	
H1	0.5344	0.0272	0.6319	0.038*	
C2	0.58540 (15)	0.1321 (3)	0.5744 (2)	0.0379 (6)	
H2	0.5820	0.0409	0.5359	0.045*	
C3	0.61944 (15)	0.2748 (3)	0.5736 (2)	0.0357 (6)	
H3	0.6373	0.2834	0.5326	0.043*	
C4	0.62620 (12)	0.4052 (3)	0.63604 (16)	0.0267 (5)	
C5	0.59552 (12)	0.3905 (3)	0.69197 (17)	0.0260 (5)	
H5	0.6002	0.4781	0.7336	0.031*	
C7	0.66029 (13)	0.6594 (3)	0.56799 (17)	0.0271 (5)	
C8	0.70977 (14)	0.7864 (3)	0.59181 (19)	0.0337 (6)	
H8	0.7493	0.8005	0.6585	0.040*	
C9	0.69953 (14)	0.8907 (3)	0.51563 (19)	0.0369 (6)	
H9	0.7322	0.9753	0.5299	0.044*	
C10	0.63992 (13)	0.8673 (3)	0.41792 (18)	0.0309 (5)	
H10	0.6330	0.9382	0.3667	0.037*	
C11	0.60197 (13)	0.6426 (3)	0.46786 (17)	0.0270 (5)	
H11	0.5691	0.5573	0.4513	0.032*	
C6	0.66989 (13)	0.5574 (3)	0.65383 (17)	0.0310 (5)	
Si1	0.5000	0.25199 (9)	0.2500	0.0252 (2)	
O1W	0.2808 (2)	0.1657 (6)	0.1183 (4)	0.168 (2)	
H1A	0.2777	0.2089	0.1676	0.252*	
H1B	0.3244	0.1856	0.1308	0.252*	

### Atomic displacement parameters (Å^2^)

**Table:**

	*U*^11^	*U*^22^	*U*^33^	*U*^12^	*U*^13^	*U*^23^
Cu1	0.0264 (2)	0.0224 (2)	0.0210 (2)	0.000	0.01529 (19)	0.000
F1	0.0410 (11)	0.0637 (13)	0.0584 (13)	−0.0046 (8)	0.0067 (10)	0.0081 (8)
F2	0.0645 (15)	0.0182 (9)	0.0684 (15)	0.000	0.0506 (13)	0.000
F3	0.1013 (17)	0.0365 (9)	0.0722 (13)	−0.0090 (8)	0.0731 (13)	−0.0082 (7)
F4	0.0658 (15)	0.0203 (9)	0.0571 (14)	0.000	0.0442 (13)	0.000
O1	0.0502 (12)	0.0604 (13)	0.0247 (10)	−0.0200 (10)	0.0126 (9)	−0.0002 (8)
N1	0.0289 (11)	0.0259 (10)	0.0233 (10)	0.0010 (7)	0.0171 (9)	0.0014 (7)
N2	0.0282 (11)	0.0240 (10)	0.0259 (10)	−0.0003 (7)	0.0160 (9)	0.0008 (7)
C1	0.0396 (14)	0.0261 (12)	0.0355 (13)	−0.0020 (10)	0.0247 (11)	−0.0023 (10)
C2	0.0507 (16)	0.0341 (13)	0.0412 (14)	−0.0035 (11)	0.0332 (13)	−0.0097 (11)
C3	0.0418 (15)	0.0406 (14)	0.0334 (14)	−0.0016 (11)	0.0264 (12)	−0.0009 (11)
C4	0.0251 (12)	0.0301 (12)	0.0221 (11)	0.0032 (9)	0.0114 (10)	0.0056 (9)
C5	0.0276 (12)	0.0264 (11)	0.0235 (11)	0.0023 (9)	0.0139 (10)	0.0010 (9)
C7	0.0287 (12)	0.0284 (11)	0.0267 (11)	−0.0012 (9)	0.0170 (10)	0.0005 (9)
C8	0.0305 (13)	0.0378 (13)	0.0284 (13)	−0.0090 (11)	0.0136 (11)	−0.0047 (10)
C9	0.0386 (14)	0.0338 (13)	0.0400 (14)	−0.0112 (11)	0.0229 (12)	−0.0023 (11)
C10	0.0372 (13)	0.0271 (11)	0.0339 (12)	−0.0031 (10)	0.0232 (11)	0.0017 (10)
C11	0.0296 (12)	0.0256 (11)	0.0278 (12)	−0.0027 (9)	0.0172 (10)	0.0010 (9)
C6	0.0284 (12)	0.0372 (13)	0.0267 (12)	−0.0014 (10)	0.0149 (11)	0.0021 (10)
Si1	0.0328 (5)	0.0179 (5)	0.0280 (5)	0.000	0.0188 (4)	0.000
O1W	0.105 (3)	0.176 (4)	0.257 (5)	−0.047 (3)	0.121 (4)	−0.145 (4)

### Geometric parameters (Å, °)

**Table:**

Cu1—N1^i^	2.033 (2)	C2—H2	0.9300
Cu1—N1^ii^	2.033 (2)	C3—C4	1.390 (3)
Cu1—N2	2.038 (2)	C3—H3	0.9300
Cu1—N2^iii^	2.038 (2)	C4—C5	1.389 (3)
Cu1—F2	2.241 (2)	C4—C6	1.493 (3)
Cu1—F4^iv^	2.401 (2)	C5—H5	0.9300
F1—Si1	1.6747 (19)	C7—C11	1.386 (3)
F2—Si1	1.714 (2)	C7—C8	1.394 (3)
F3—Si1	1.6636 (17)	C7—C6	1.494 (3)
F4—Si1	1.707 (2)	C8—C9	1.378 (4)
F4—Cu1^v^	2.401 (2)	C8—H8	0.9300
O1—C6	1.212 (3)	C9—C10	1.378 (4)
N1—C5	1.342 (3)	C9—H9	0.9300
N1—C1	1.352 (3)	C10—H10	0.9300
N1—Cu1^ii^	2.033 (2)	C11—H11	0.9300
N2—C10	1.341 (3)	Si1—F3^iii^	1.6636 (17)
N2—C11	1.351 (3)	Si1—F1^iii^	1.6747 (19)
C1—C2	1.371 (4)	O1W—H1A	0.8900
C1—H1	0.9300	O1W—H1B	0.8901
C2—C3	1.382 (4)		
			
N1^i^—Cu1—N1^ii^	178.65 (9)	N1—C5—H5	118.8
N1^i^—Cu1—N2	91.03 (8)	C4—C5—H5	118.8
N1^ii^—Cu1—N2	88.95 (8)	C11—C7—C8	118.6 (2)
N1^i^—Cu1—N2^iii^	88.95 (8)	C11—C7—C6	123.4 (2)
N1^ii^—Cu1—N2^iii^	91.03 (8)	C8—C7—C6	117.8 (2)
N2—Cu1—N2^iii^	178.15 (9)	C9—C8—C7	119.4 (2)
N1^i^—Cu1—F2	90.68 (5)	C9—C8—H8	120.3
N1^ii^—Cu1—F2	90.68 (5)	C7—C8—H8	120.3
N2—Cu1—F2	90.93 (5)	C8—C9—C10	118.8 (2)
N2^iii^—Cu1—F2	90.93 (5)	C8—C9—H9	120.6
N1^i^—Cu1—F4^iv^	89.32 (5)	C10—C9—H9	120.6
N1^ii^—Cu1—F4^iv^	89.32 (5)	N2—C10—C9	122.8 (2)
N2—Cu1—F4^iv^	89.07 (5)	N2—C10—H10	118.6
N2^iii^—Cu1—F4^iv^	89.07 (5)	C9—C10—H10	118.6
F2—Cu1—F4^iv^	180.0	N2—C11—C7	121.9 (2)
Si1—F2—Cu1	180.000 (1)	N2—C11—H11	119.1
Si1—F4—Cu1^v^	180.000 (1)	C7—C11—H11	119.1
C5—N1—C1	117.9 (2)	O1—C6—C4	118.3 (2)
C5—N1—Cu1^ii^	120.21 (15)	O1—C6—C7	119.5 (2)
C1—N1—Cu1^ii^	121.42 (16)	C4—C6—C7	122.1 (2)
C10—N2—C11	118.6 (2)	F3—Si1—F3^iii^	179.09 (11)
C10—N2—Cu1	118.57 (16)	F3—Si1—F1^iii^	89.62 (12)
C11—N2—Cu1	122.52 (15)	F3^iii^—Si1—F1^iii^	90.36 (12)
N1—C1—C2	122.3 (2)	F3—Si1—F1	90.36 (12)
N1—C1—H1	118.8	F3^iii^—Si1—F1	89.62 (12)
C2—C1—H1	118.8	F1^iii^—Si1—F1	178.52 (13)
C1—C2—C3	120.2 (2)	F3—Si1—F4	90.45 (5)
C1—C2—H2	119.9	F3^iii^—Si1—F4	90.45 (5)
C3—C2—H2	119.9	F1^iii^—Si1—F4	90.74 (6)
C2—C3—C4	117.8 (2)	F1—Si1—F4	90.74 (6)
C2—C3—H3	121.1	F3—Si1—F2	89.55 (5)
C4—C3—H3	121.1	F3^iii^—Si1—F2	89.55 (5)
C5—C4—C3	119.2 (2)	F1^iii^—Si1—F2	89.26 (6)
C5—C4—C6	116.8 (2)	F1—Si1—F2	89.26 (6)
C3—C4—C6	123.9 (2)	F4—Si1—F2	180.0
N1—C5—C4	122.4 (2)	H1A—O1W—H1B	109.8
			
N1^i^—Cu1—N2—C10	54.16 (18)	C11—C7—C8—C9	−0.2 (4)
N1^ii^—Cu1—N2—C10	−124.49 (18)	C6—C7—C8—C9	−175.2 (2)
F2—Cu1—N2—C10	144.85 (17)	C7—C8—C9—C10	0.7 (4)
F4^iv^—Cu1—N2—C10	−35.15 (17)	C11—N2—C10—C9	−0.6 (4)
N1^i^—Cu1—N2—C11	−132.42 (18)	Cu1—N2—C10—C9	173.0 (2)
N1^ii^—Cu1—N2—C11	48.93 (18)	C8—C9—C10—N2	−0.3 (4)
F2—Cu1—N2—C11	−41.73 (17)	C10—N2—C11—C7	1.2 (3)
F4^iv^—Cu1—N2—C11	138.27 (17)	Cu1—N2—C11—C7	−172.22 (17)
C5—N1—C1—C2	−4.1 (4)	C8—C7—C11—N2	−0.8 (4)
Cu1^ii^—N1—C1—C2	167.9 (2)	C6—C7—C11—N2	173.9 (2)
N1—C1—C2—C3	0.8 (4)	C5—C4—C6—O1	45.3 (3)
C1—C2—C3—C4	3.0 (4)	C3—C4—C6—O1	−129.8 (3)
C2—C3—C4—C5	−3.4 (4)	C5—C4—C6—C7	−132.4 (2)
C2—C3—C4—C6	171.6 (2)	C3—C4—C6—C7	52.6 (3)
C1—N1—C5—C4	3.7 (3)	C11—C7—C6—O1	−164.2 (2)
Cu1^ii^—N1—C5—C4	−168.43 (17)	C8—C7—C6—O1	10.6 (4)
C3—C4—C5—N1	0.0 (3)	C11—C7—C6—C4	13.4 (4)
C6—C4—C5—N1	−175.3 (2)	C8—C7—C6—C4	−171.8 (2)

Symmetry codes: (i) *x*, −*y*+1, *z*−1/2; (ii) −*x*+1, −*y*+1, −*z*+1; (iii) −*x*+1, *y*, −*z*+1/2; (iv) *x*, *y*+1, *z*; (v) *x*, *y*−1, *z*.

### Hydrogen-bond geometry (Å, °)

**Table:**

*D*—H···*A*	*D*—H	H···*A*	*D*···*A*	*D*—H···*A*
O1W—H1B···F1	0.89	1.89	2.777 (6)	173
O1W—H1A···O1^ii^	0.89	2.03	2.850 (3)	153

Symmetry codes: (ii) −*x*+1, −*y*+1, −*z*+1.
